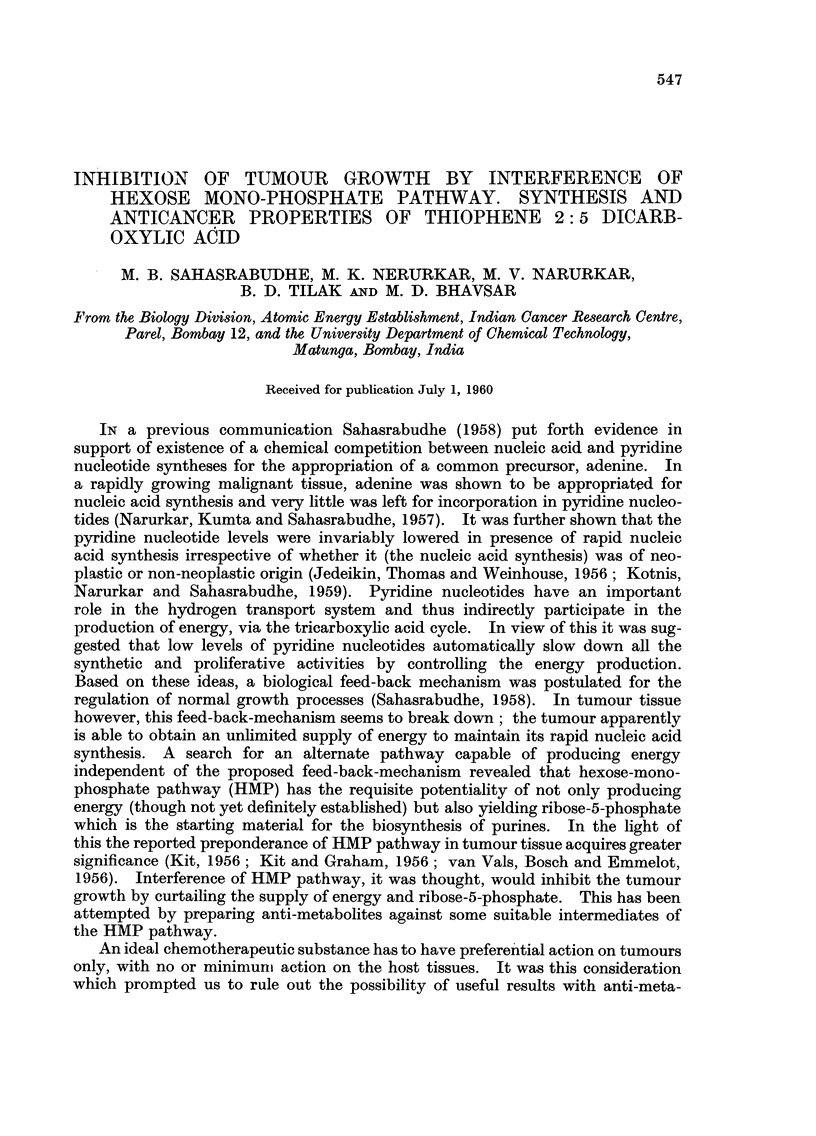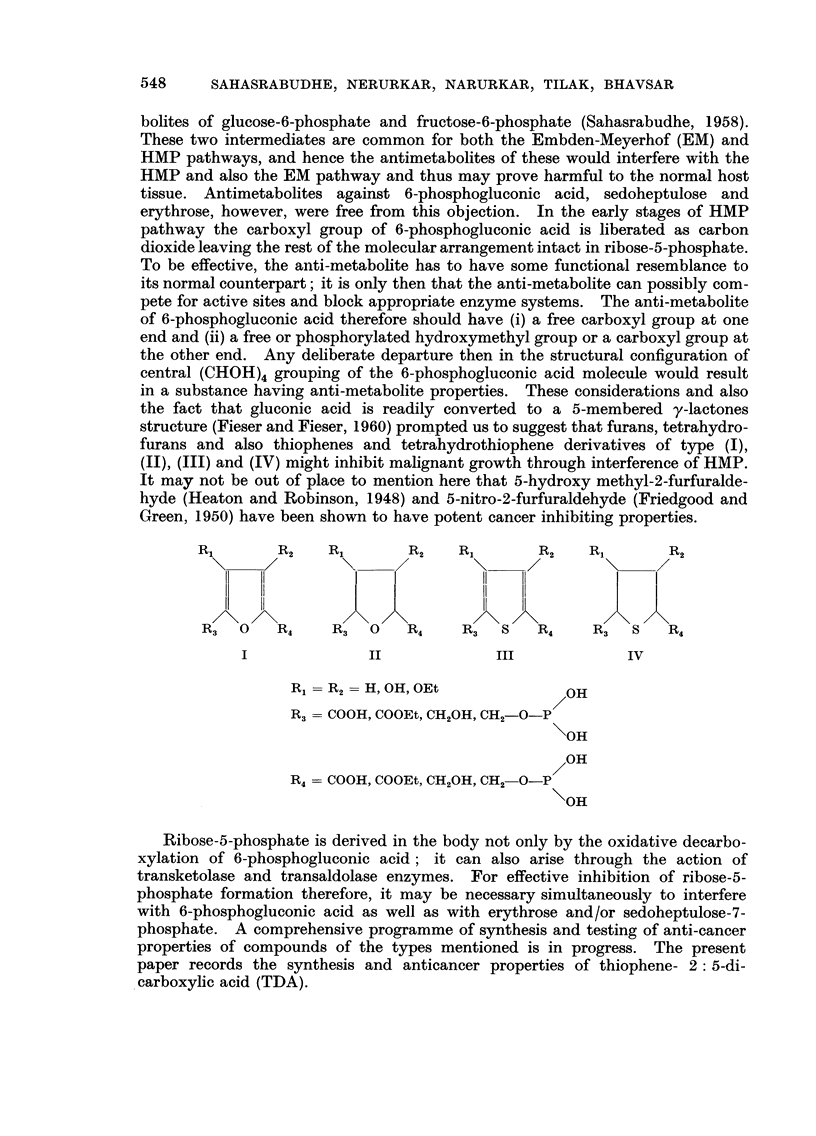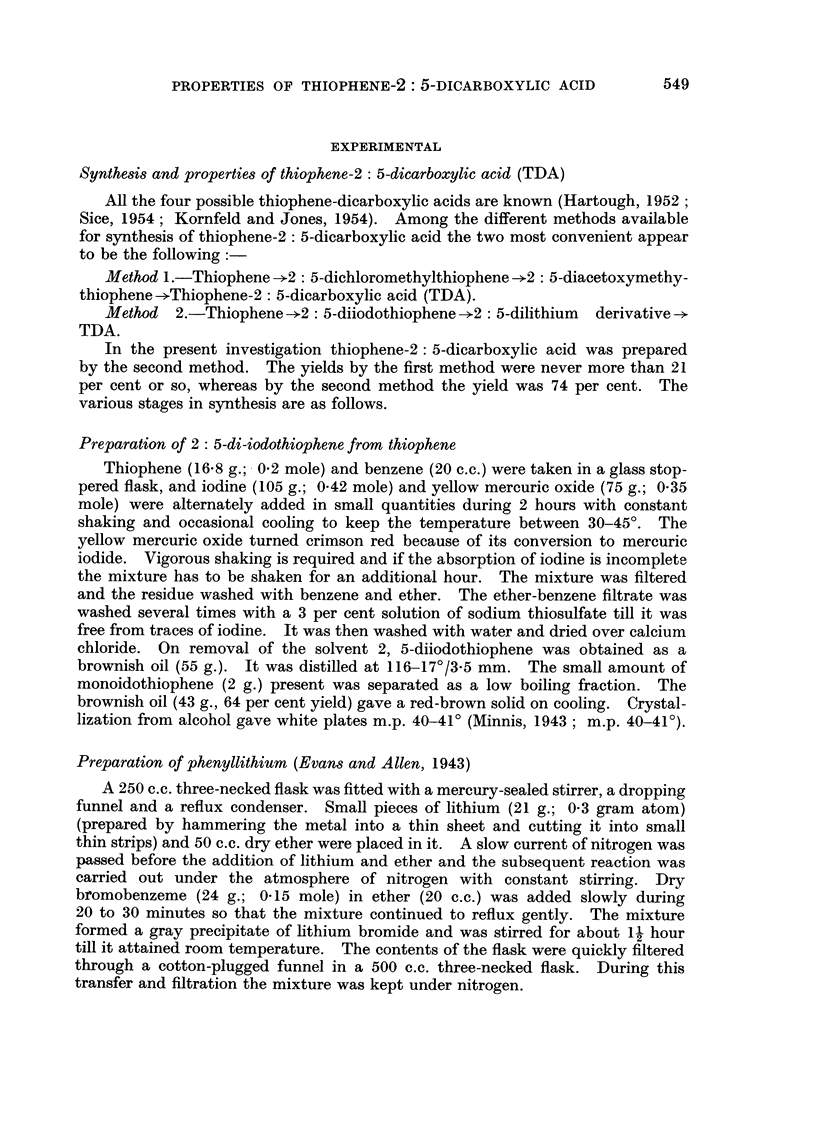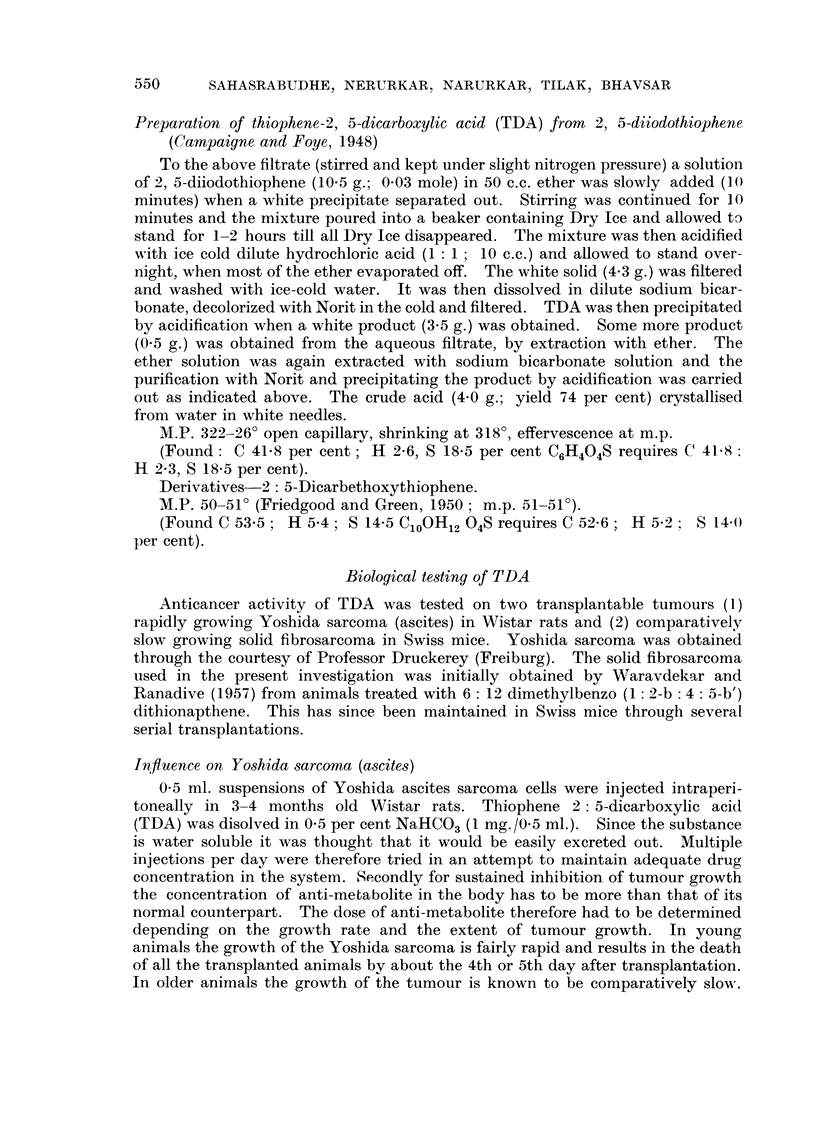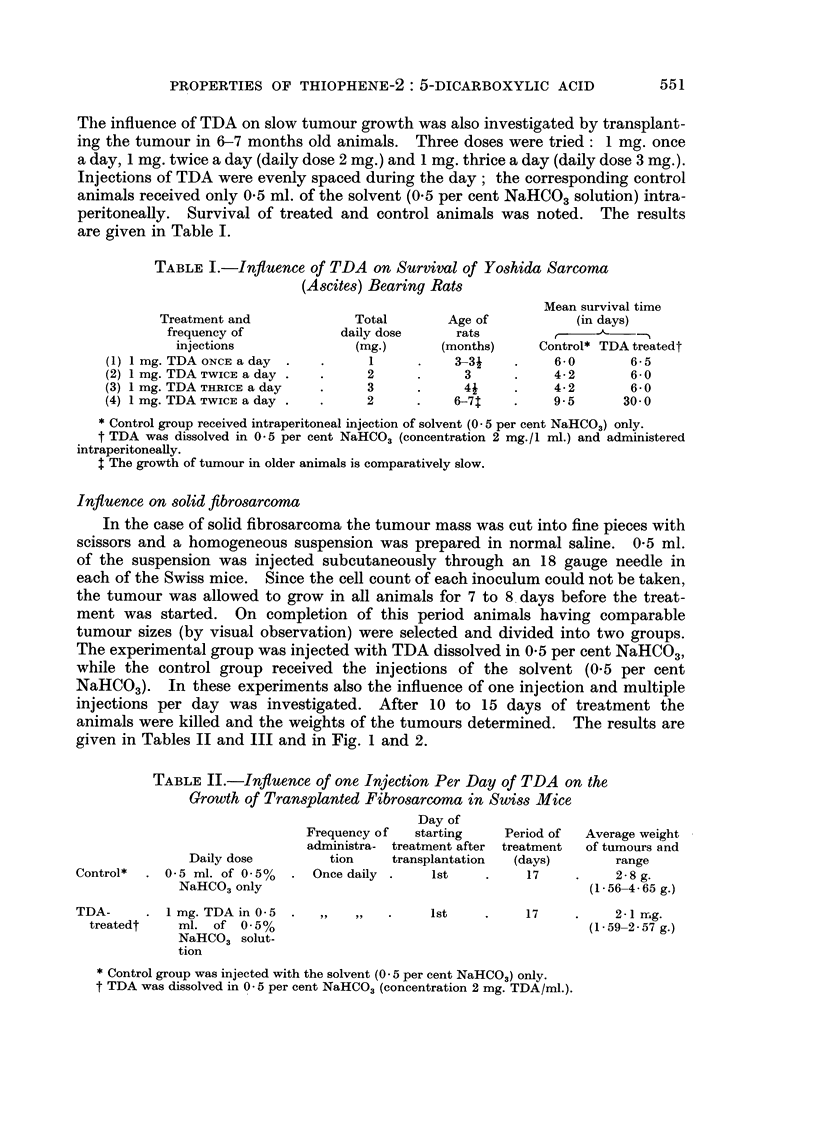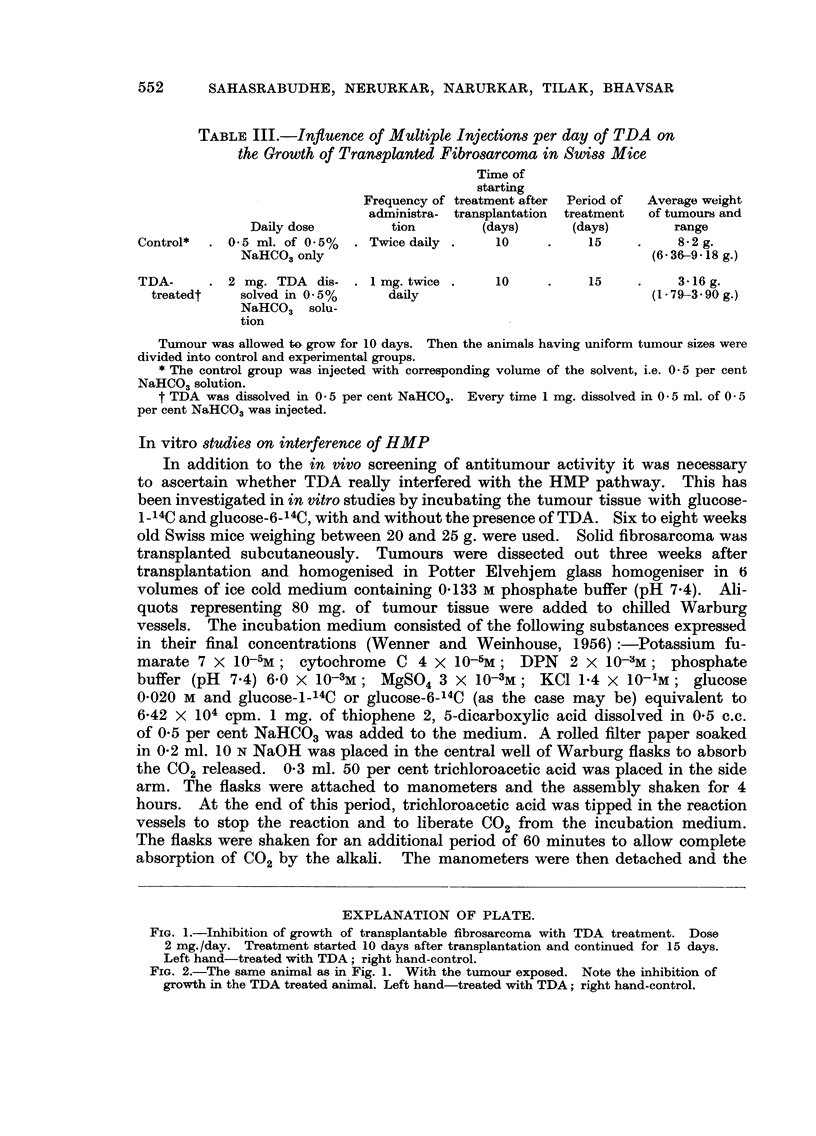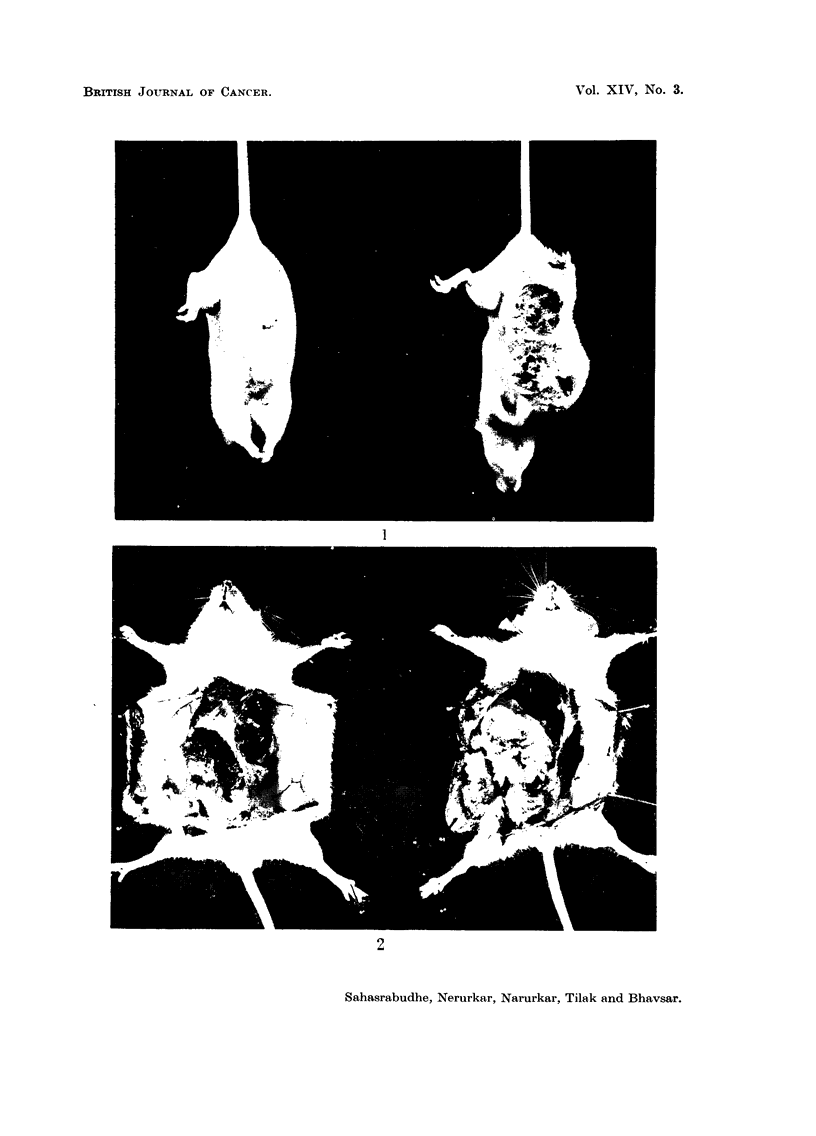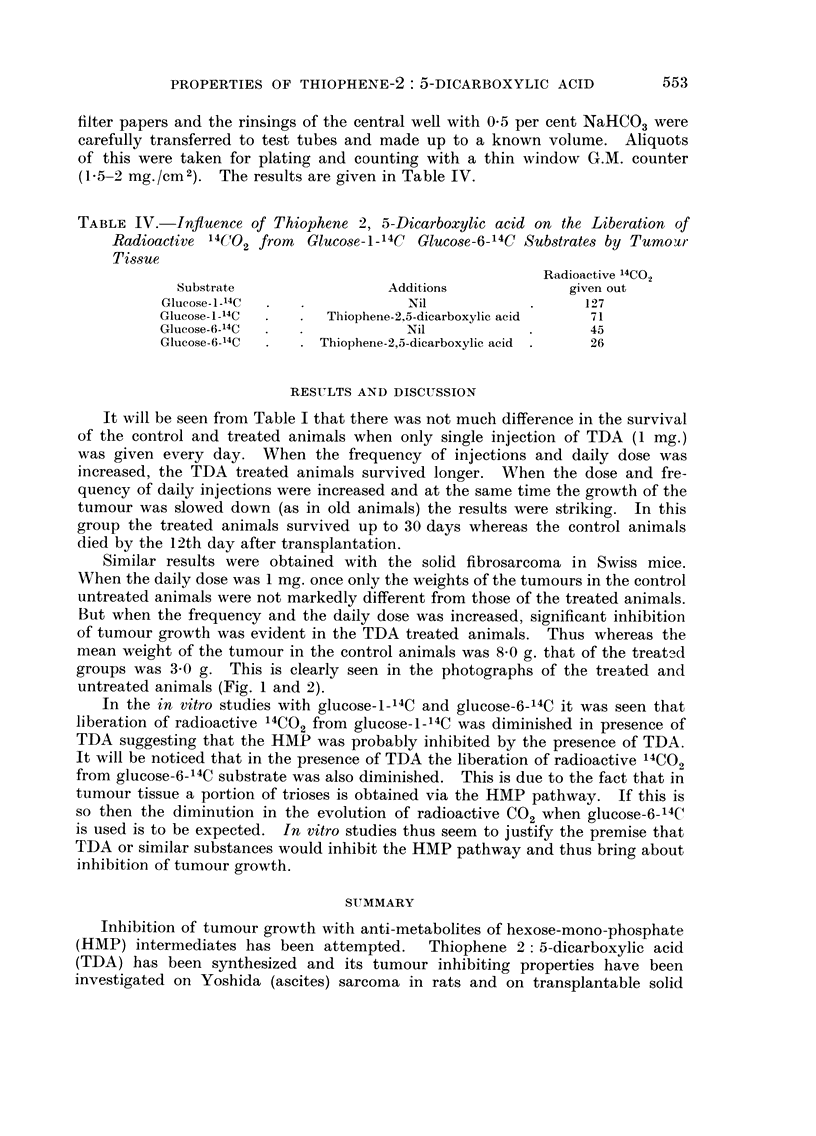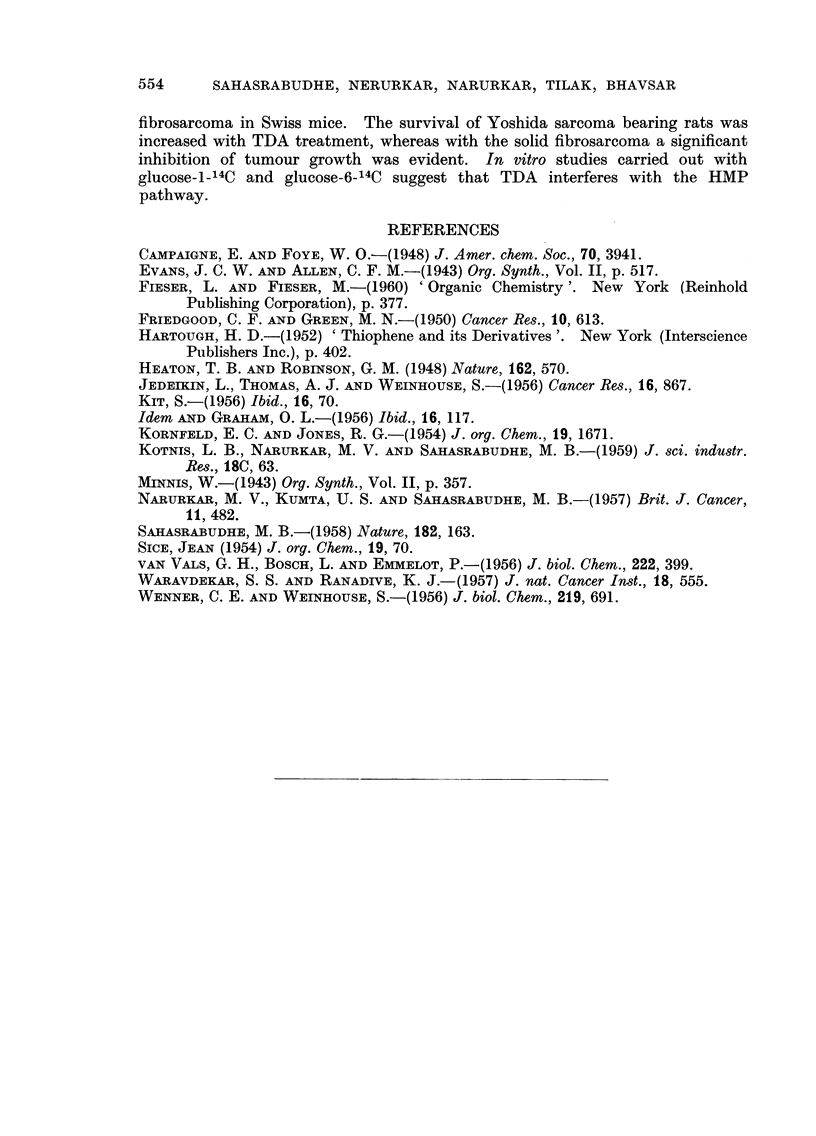# Inhibition of Tumour Growth by Interference of Hexose-mono-phosphate Pathway. Synthesis and Anticancer Properties of Thiophene 2:5 Dicarboxylic Acid

**DOI:** 10.1038/bjc.1960.61

**Published:** 1960-09

**Authors:** M. B. Sahasrabudhe, M. K. Nerurkar, M. V. Narurkar, B. D. Tilak, M. D. Bhavsar

## Abstract

**Images:**


### INHIBITION OF TUMOUR GROWTH BY INTERFERENCE OF HEXOSE MONO-PHOSPHATE PATHWAY. SYNTHESIS AND ANTICANCER PROPERTIES OF THIOPHENE 2 : 5 DICARBOXYLIC ACID

M. B. SAHASRABUDHE, M. K. NERURKAR, M. V. NARURKAR, B. D. TILAK AND M. D. BHAVSAR

*From the Biology Division, Atomic Energy Establishment, Indian Cancer Research Centre, Parel, Bombay 12, and the University Department of Chemical Technology, Matunga, Bombay, India*

Received for publication July 1, 1960

In a previous communication Sahasrabudhe (1958) put forth evidence in support of existence of a chemical competition between nucleic acid and pyridine nucleotide syntheses for the appropriation of a common precursor, adenine. In a rapidly growing malignant tissue, adenine was shown to be appropriated for nucleic acid synthesis and very little was left for incorporation in pyridine nucleotides (Narurkar, Kumta and Sahasrabudhe, 1957). It was further shown that the pyridine nucleotide levels were invariably lowered in presence of rapid nucleic acid synthesis irrespective of whether it (the nucleic acid synthesis) was of neoplastic or non-neoplastic origin (Jedeikin, Thomas and Weinhouse, 1956 ; Kotnis, Narurkar and Sahasrabudhe, 1959). Pyridine nucleotides have an important role in the hydrogen transport system and thus indirectly participate in the production of energy, via the tricarboxylic acid cycle. In view of this it was suggested that low levels of pyridine nucleotides automatically slow down all the synthetic and proliferative activities by controlling the energy production. Based on these ideas, a biological feed-back mechanism was postulated for the regulation of normal growth processes (Sahasrabudhe, 1958). In tumour tissue however, this feed-back-mechanism seems to break down ; the tumour apparently is able to obtain an unlimited supply of energy to maintain its rapid nucleic acid synthesis. A search for an alternate pathway capable of producing energy independent of the proposed feed-back-mechanism revealed that hexose-mono-phosphate pathway (HMP) has the requisite potentiality of not only producing energy (though not yet definitely established) but also yielding ribose-5-phosphate which is the starting material for the biosynthesis of purines. In the light of this the reported preponderance of HMP pathway in tumour tissue acquires greater significance (Kit, 1956 ; Kit and Graham, 1956 ; van Vals, Bosch and Emmelot, 1956). Interference of HMP pathway, it was thought, would inhibit the tumour growth by curtailing the supply of energy and ribose-5-phosphate. This has been attempted by preparing anti-metabolites against some suitable intermediates of the HMP pathway.

An ideal chemotherapeutic substance has to have preferential action on tumours only, with no or minimum action on the host tissues. It was this consideration which prompted us to rule out the possibility of useful results with anti-metabolites of glucose-6-phosphate and fructose-6-phosphate (Sahasrabudhe, 1958). These two intermediates are common for both the Embden-Meyerhof (EM) and HMP pathways, and hence the antimetabolites of these would interfere with the HMP and also the EM pathway and thus may prove harmful to the normal host tissue. Antimetabolites against 6-phosphogluconic acid, sedoheptulose and erythrose, however, were free from this objection. In the early stages of HMP pathway the carboxyl group of 6-phosphogluconic acid is liberated as carbon dioxide leaving the rest of the molecular arrangement intact in ribose-5-phosphate. To be effective, the anti-metabolite has to have some functional resemblance to its normal counterpart; it is only then that the anti-metabolite can possibly compete for active sites and block appropriate enzyme systems. The anti-metabolite of 6-phosphogluconic acid therefore should have (i) a free carboxyl group at one end and (ii) a free or phosphorylated hydroxymethyl group or a carboxyl group at the other end. Any deliberate departure then in the structural configuration of central $(CHOH)_4$ grouping of the 6-phosphogluconic acid molecule would result in a substance having anti-metabolite properties. These considerations and also the fact that gluconic acid is readily converted to a 5-membered $\gamma$-lactones structure (Fieser and Fieser, 1960) prompted us to suggest that furans, tetrahydrofurans and also thiophenes and tetrahydrothiophene derivatives of type (I), (II), (III) and (IV) might inhibit malignant growth through interference of HMP. It may not be out of place to mention here that 5-hydroxy methyl-2-furfuraldehyde (Heaton and Robinson, 1948) and 5-nitro-2-furfuraldehyde (Friedgood and Green, 1950) have been shown to have potent cancer inhibiting properties.

I

II

III

IV

$R_1 = R_2 = H, OH, OEt$

$R_3 = COOH, COOEt, CH_2OH, CH_2—O—P(OH)_2$

$R_4 = COOH, COOEt, CH_2OH, CH_2—O—P(OH)_2$

Ribose-5-phosphate is derived in the body not only by the oxidative decarboxylation of 6-phosphogluconic acid; it can also arise through the action of transketolase and transaldolase enzymes. For effective inhibition of ribose-5-phosphate formation therefore, it may be necessary simultaneously to interfere with 6-phosphogluconic acid as well as with erythrose and/or sedoheptulose-7-phosphate. A comprehensive programme of synthesis and testing of anti-cancer properties of compounds of the types mentioned is in progress. The present paper records the synthesis and anticancer properties of thiophene- 2 : 5-dicarboxylic acid (TDA).

### EXPERIMENTAL

### *Synthesis and properties of thiophene-2 : 5-dicarboxylic acid* (TDA)

All the four possible thiophene-dicarboxylic acids are known (Hartough, 1952 ; Sice, 1954 ; Kornfeld and Jones, 1954). Among the different methods available for synthesis of thiophene-2 : 5-dicarboxylic acid the two most convenient appear to be the following :—

*Method* 1.—Thiophene→2 : 5-dichloromethylthiophene→2 : 5-diacetoxymethythiophene→Thiophene-2 : 5-dicarboxylic acid (TDA).

*Method* 2.—Thiophene→2 : 5-diiodothiophene→2 : 5-dilithium derivative→ TDA.

In the present investigation thiophene-2 : 5-dicarboxylic acid was prepared by the second method. The yields by the first method were never more than 21 per cent or so, whereas by the second method the yield was 74 per cent. The various stages in synthesis are as follows.

### *Preparation of 2 : 5-di-iodothiophene from thiophene*

Thiophene (16·8 g.; 0·2 mole) and benzene (20 c.c.) were taken in a glass stoppered flask, and iodine (105 g.; 0·42 mole) and yellow mercuric oxide (75 g.; 0·35 mole) were alternately added in small quantities during 2 hours with constant shaking and occasional cooling to keep the temperature between 30–45°. The yellow mercuric oxide turned crimson red because of its conversion to mercuric iodide. Vigorous shaking is required and if the absorption of iodine is incomplete the mixture has to be shaken for an additional hour. The mixture was filtered and the residue washed with benzene and ether. The ether-benzene filtrate was washed several times with a 3 per cent solution of sodium thiosulfate till it was free from traces of iodine. It was then washed with water and dried over calcium chloride. On removal of the solvent 2, 5-diiodothiophene was obtained as a brownish oil (55 g.). It was distilled at 116–17°/3·5 mm. The small amount of monoidothiophene (2 g.) present was separated as a low boiling fraction. The brownish oil (43 g., 64 per cent yield) gave a red-brown solid on cooling. Crystallization from alcohol gave white plates m.p. 40–41° (Minnis, 1943 ; m.p. 40–41°).

### *Preparation of phenyllithium* (*Evans and Allen*, 1943)

A 250 c.c. three-necked flask was fitted with a mercury-sealed stirrer, a dropping funnel and a reflux condenser. Small pieces of lithium (21 g.; 0·3 gram atom) (prepared by hammering the metal into a thin sheet and cutting it into small thin strips) and 50 c.c. dry ether were placed in it. A slow current of nitrogen was passed before the addition of lithium and ether and the subsequent reaction was carried out under the atmosphere of nitrogen with constant stirring. Dry bromobenzeme (24 g.; 0·15 mole) in ether (20 c.c.) was added slowly during 20 to 30 minutes so that the mixture continued to reflux gently. The mixture formed a gray precipitate of lithium bromide and was stirred for about 1½ hour till it attained room temperature. The contents of the flask were quickly filtered through a cotton-plugged funnel in a 500 c.c. three-necked flask. During this transfer and filtration the mixture was kept under nitrogen.

*Preparation of thiophene-2, 5-dicarboxylic acid* (TDA) *from* 2, *5-diiodothiophene* (*Campaigne and Foye*, 1948)

To the above filtrate (stirred and kept under slight nitrogen pressure) a solution of 2, 5-diiodothiophene (10·5 g.; 0·03 mole) in 50 c.c. ether was slowly added (10 minutes) when a white precipitate separated out. Stirring was continued for 10 minutes and the mixture poured into a beaker containing Dry Ice and allowed to stand for 1–2 hours till all Dry Ice disappeared. The mixture was then acidified with ice cold dilute hydrochloric acid (1 : 1 ; 10 c.c.) and allowed to stand overnight, when most of the ether evaporated off. The white solid (4·3 g.) was filtered and washed with ice-cold water. It was then dissolved in dilute sodium bicarbonate, decolorized with Norit in the cold and filtered. TDA was then precipitated by acidification when a white product (3·5 g.) was obtained. Some more product (0·5 g.) was obtained from the aqueous filtrate, by extraction with ether. The ether solution was again extracted with sodium bicarbonate solution and the purification with Norit and precipitating the product by acidification was carried out as indicated above. The crude acid (4·0 g.; yield 74 per cent) crystallised from water in white needles.

M.P. 322–26° open capillary, shrinking at 318°, effervescence at m.p.

(Found : C 41·8 per cent ; H 2·6, S 18·5 per cent $C_6H_4O_4S$ requires C 41·8 ; H 2·3, S 18·5 per cent).

Derivatives—2 : 5-Dicarbethoxythiophene.

M.P. 50–51° (Friedgood and Green, 1950 ; m.p. 51–51°).

(Found C 53·5 ; H 5·4 ; S 14·5 $C_{10}OH_{12}$ $O_4S$ requires C 52·6 ; H 5·2 ; S 14·0 per cent).

*Biological testing of TDA*

Anticancer activity of TDA was tested on two transplantable tumours (1) rapidly growing Yoshida sarcoma (ascites) in Wistar rats and (2) comparatively slow growing solid fibrosarcoma in Swiss mice. Yoshida sarcoma was obtained through the courtesy of Professor Druckerey (Freiburg). The solid fibrosarcoma used in the present investigation was initially obtained by Waravdekar and Ranadive (1957) from animals treated with 6 : 12 dimethylbenzo (1 : 2-b : 4 : 5-b′) dithionapthene. This has since been maintained in Swiss mice through several serial transplantations.

*Influence on Yoshida sarcoma* (*ascites*)

0·5 ml. suspensions of Yoshida ascites sarcoma cells were injected intraperitoneally in 3–4 months old Wistar rats. Thiophene 2 : 5-dicarboxylic acid (TDA) was disolved in 0·5 per cent $NaHCO_3$ (1 mg./0·5 ml.). Since the substance is water soluble it was thought that it would be easily excreted out. Multiple injections per day were therefore tried in an attempt to maintain adequate drug concentration in the system. Secondly for sustained inhibition of tumour growth the concentration of anti-metabolite in the body has to be more than that of its normal counterpart. The dose of anti-metabolite therefore had to be determined depending on the growth rate and the extent of tumour growth. In young animals the growth of the Yoshida sarcoma is fairly rapid and results in the death of all the transplanted animals by about the 4th or 5th day after transplantation. In older animals the growth of the tumour is known to be comparatively slow.

The influence of TDA on slow tumour growth was also investigated by transplanting the tumour in 6–7 months old animals. Three doses were tried : 1 mg. once a day, 1 mg. twice a day (daily dose 2 mg.) and 1 mg. thrice a day (daily dose 3 mg.). Injections of TDA were evenly spaced during the day ; the corresponding control animals received only 0·5 ml. of the solvent (0·5 per cent $NaHCO_3$ solution) intraperitoneally. Survival of treated and control animals was noted. The results are given in Table I.

TABLE I.—*Influence of TDA on Survival of Yoshida Sarcoma (Ascites) Bearing Rats*

| Treatment and frequency of injections | Total daily dose (mg.) | Age of rats (months) | Mean survival time (in days) Control* | TDA treated† |
|---|---|---|---|---|
| (1) 1 mg. TDA ONCE a day | 1 | 3–3½ | 6·0 | 6·5 |
| (2) 1 mg. TDA TWICE a day | 2 | 3 | 4·2 | 6·0 |
| (3) 1 mg. TDA THRICE a day | 3 | 4½ | 4·2 | 6·0 |
| (4) 1 mg. TDA TWICE a day | 2 | 6–7‡ | 9·5 | 30·0 |

* Control group received intraperitoneal injection of solvent (0·5 per cent $NaHCO_3$) only.
† TDA was dissolved in 0·5 per cent $NaHCO_3$ (concentration 2 mg./1 ml.) and administered intraperitoneally.
‡ The growth of tumour in older animals is comparatively slow.

*Influence on solid fibrosarcoma*

In the case of solid fibrosarcoma the tumour mass was cut into fine pieces with scissors and a homogeneous suspension was prepared in normal saline. 0·5 ml. of the suspension was injected subcutaneously through an 18 gauge needle in each of the Swiss mice. Since the cell count of each inoculum could not be taken, the tumour was allowed to grow in all animals for 7 to 8 days before the treatment was started. On completion of this period animals having comparable tumour sizes (by visual observation) were selected and divided into two groups. The experimental group was injected with TDA dissolved in 0·5 per cent $NaHCO_3$, while the control group received the injections of the solvent (0·5 per cent $NaHCO_3$). In these experiments also the influence of one injection and multiple injections per day was investigated. After 10 to 15 days of treatment the animals were killed and the weights of the tumours determined. The results are given in Tables II and III and in Fig. 1 and 2.

TABLE II.—*Influence of one Injection Per Day of TDA on the Growth of Transplanted Fibrosarcoma in Swiss Mice*

| | Daily dose | Frequency of administration | Day of starting treatment after transplantation | Period of treatment (days) | Average weight of tumours and range |
|---|---|---|---|---|---|
| Control* | 0·5 ml. of 0·5% $NaHCO_3$ only | Once daily | 1st | 17 | 2·8 g. (1·56–4·65 g.) |
| TDA-treated† | 1 mg. TDA in 0·5 ml. of 0·5% $NaHCO_3$ solution | „ „ | 1st | 17 | 2·1 mg. (1·59–2·57 g.) |

* Control group was injected with the solvent (0·5 per cent $NaHCO_3$) only.
† TDA was dissolved in 0·5 per cent $NaHCO_3$ (concentration 2 mg. TDA/ml.).

TABLE III.—*Influence of Multiple Injections per day of TDA on the Growth of Transplanted Fibrosarcoma in Swiss Mice*

| | Daily dose | Frequency of administration | Time of starting treatment after transplantation (days) | Period of treatment (days) | Average weight of tumours and range |
|---|---|---|---|---|---|
| Control* | 0·5 ml. of 0·5% $NaHCO_3$ only | Twice daily | 10 | 15 | 8·2 g. (6·36–9·18 g.) |
| TDA-treated† | 2 mg. TDA dissolved in 0·5% $NaHCO_3$ solution | 1 mg. twice daily | 10 | 15 | 3·16 g. (1·79–3·90 g.) |

Tumour was allowed to grow for 10 days. Then the animals having uniform tumour sizes were divided into control and experimental groups.

* The control group was injected with corresponding volume of the solvent, i.e. 0·5 per cent $NaHCO_3$ solution.

† TDA was dissolved in 0·5 per cent $NaHCO_3$. Every time 1 mg. dissolved in 0·5 ml. of 0·5 per cent $NaHCO_3$ was injected.

### In vitro *studies on interference of HMP*

In addition to the *in vivo* screening of antitumour activity it was necessary to ascertain whether TDA really interfered with the HMP pathway. This has been investigated in *in vitro* studies by incubating the tumour tissue with glucose-1-$^{14}C$ and glucose-6-$^{14}C$, with and without the presence of TDA. Six to eight weeks old Swiss mice weighing between 20 and 25 g. were used. Solid fibrosarcoma was transplanted subcutaneously. Tumours were dissected out three weeks after transplantation and homogenised in Potter Elvehjem glass homogeniser in 6 volumes of ice cold medium containing 0·133 M phosphate buffer (pH 7·4). Aliquots representing 80 mg. of tumour tissue were added to chilled Warburg vessels. The incubation medium consisted of the following substances expressed in their final concentrations (Wenner and Weinhouse, 1956) :—Potassium fumarate $7 \times 10^{-5}$M ; cytochrome C $4 \times 10^{-5}$M ; DPN $2 \times 10^{-3}$M ; phosphate buffer (pH 7·4) $6{\cdot}0 \times 10^{-3}$M ; $MgSO_4$ $3 \times 10^{-3}$M ; KCl $1{\cdot}4 \times 10^{-1}$M ; glucose 0·020 M and glucose-1-$^{14}C$ or glucose-6-$^{14}C$ (as the case may be) equivalent to $6{\cdot}42 \times 10^4$ cpm. 1 mg. of thiophene 2, 5-dicarboxylic acid dissolved in 0·5 c.c. of 0·5 per cent $NaHCO_3$ was added to the medium. A rolled filter paper soaked in 0·2 ml. 10 N NaOH was placed in the central well of Warburg flasks to absorb the $CO_2$ released. 0·3 ml. 50 per cent trichloroacetic acid was placed in the side arm. The flasks were attached to manometers and the assembly shaken for 4 hours. At the end of this period, trichloroacetic acid was tipped in the reaction vessels to stop the reaction and to liberate $CO_2$ from the incubation medium. The flasks were shaken for an additional period of 60 minutes to allow complete absorption of $CO_2$ by the alkali. The manometers were then detached and the

EXPLANATION OF PLATE.

FIG. 1.—Inhibition of growth of transplantable fibrosarcoma with TDA treatment. Dose 2 mg./day. Treatment started 10 days after transplantation and continued for 15 days. Left hand—treated with TDA ; right hand-control.

FIG. 2.—The same animal as in Fig. 1. With the tumour exposed. Note the inhibition of growth in the TDA treated animal. Left hand—treated with TDA ; right hand-control.

1

2

Sahasrabudhe, Nerurkar, Narurkar, Tilak and Bhavsar.

filter papers and the rinsings of the central well with 0·5 per cent $NaHCO_3$ were carefully transferred to test tubes and made up to a known volume. Aliquots of this were taken for plating and counting with a thin window G.M. counter (1·5–2 mg./cm$^2$). The results are given in Table IV.

TABLE IV.—*Influence of Thiophene 2, 5-Dicarboxylic acid on the Liberation of Radioactive* $^{14}CO_2$ *from Glucose-1-*$^{14}C$ *Glucose-6-*$^{14}C$ *Substrates by Tumour Tissue*

| Substrate | Additions | Radioactive $^{14}CO_2$ given out |
|---|---|---|
| Glucose-1-$^{14}C$ | Nil | 127 |
| Glucose-1-$^{14}C$ | Thiophene-2,5-dicarboxylic acid | 71 |
| Glucose-6-$^{14}C$ | Nil | 45 |
| Glucose-6-$^{14}C$ | Thiophene-2,5-dicarboxylic acid | 26 |

### RESULTS AND DISCUSSION

It will be seen from Table I that there was not much difference in the survival of the control and treated animals when only single injection of TDA (1 mg.) was given every day. When the frequency of injections and daily dose was increased, the TDA treated animals survived longer. When the dose and frequency of daily injections were increased and at the same time the growth of the tumour was slowed down (as in old animals) the results were striking. In this group the treated animals survived up to 30 days whereas the control animals died by the 12th day after transplantation.

Similar results were obtained with the solid fibrosarcoma in Swiss mice. When the daily dose was 1 mg. once only the weights of the tumours in the control untreated animals were not markedly different from those of the treated animals. But when the frequency and the daily dose was increased, significant inhibition of tumour growth was evident in the TDA treated animals. Thus whereas the mean weight of the tumour in the control animals was 8·0 g. that of the treated groups was 3·0 g. This is clearly seen in the photographs of the treated and untreated animals (Fig. 1 and 2).

In the *in vitro* studies with glucose-1-$^{14}C$ and glucose-6-$^{14}C$ it was seen that liberation of radioactive $^{14}CO_2$ from glucose-1-$^{14}C$ was diminished in presence of TDA suggesting that the HMP was probably inhibited by the presence of TDA. It will be noticed that in the presence of TDA the liberation of radioactive $^{14}CO_2$ from glucose-6-$^{14}C$ substrate was also diminished. This is due to the fact that in tumour tissue a portion of trioses is obtained via the HMP pathway. If this is so then the diminution in the evolution of radioactive $CO_2$ when glucose-6-$^{14}C$ is used is to be expected. *In vitro* studies thus seem to justify the premise that TDA or similar substances would inhibit the HMP pathway and thus bring about inhibition of tumour growth.

### SUMMARY

Inhibition of tumour growth with anti-metabolites of hexose-mono-phosphate (HMP) intermediates has been attempted. Thiophene 2 : 5-dicarboxylic acid (TDA) has been synthesized and its tumour inhibiting properties have been investigated on Yoshida (ascites) sarcoma in rats and on transplantable solid fibrosarcoma in Swiss mice. The survival of Yoshida sarcoma bearing rats was increased with TDA treatment, whereas with the solid fibrosarcoma a significant inhibition of tumour growth was evident. *In vitro* studies carried out with glucose-1-$^{14}C$ and glucose-6-$^{14}C$ suggest that TDA interferes with the HMP pathway.

### REFERENCES

CAMPAIGNE, E. AND FOYE, W. O.—(1948) *J. Amer. chem. Soc.*, **70**, 3941.
EVANS, J. C. W. AND ALLEN, C. F. M.—(1943) *Org. Synth.*, Vol. II, p. 517.
FIESER, L. AND FIESER, M.—(1960) ' Organic Chemistry '. New York (Reinhold Publishing Corporation), p. 377.
FRIEDGOOD, C. F. AND GREEN, M. N.—(1950) *Cancer Res.*, **10**, 613.
HARTOUGH, H. D.—(1952) ' Thiophene and its Derivatives '. New York (Interscience Publishers Inc.), p. 402.
HEATON, T. B. AND ROBINSON, G. M. (1948) *Nature*, **162**, 570.
JEDEIKIN, L., THOMAS, A. J. AND WEINHOUSE, S.—(1956) *Cancer Res.*, **16**, 867.
KIT, S.—(1956) *Ibid.*, **16**, 70.
*Idem* AND GRAHAM, O. L.—(1956) *Ibid.*, **16**, 117.
KORNFELD, E. C. AND JONES, R. G.—(1954) *J. org. Chem.*, **19**, 1671.
KOTNIS, L. B., NARURKAR, M. V. AND SAHASRABUDHE, M. B.—(1959) *J. sci. industr. Res.*, **18**C, 63.
MINNIS, W.—(1943) *Org. Synth.*, Vol. II, p. 357.
NARURKAR, M. V., KUMTA, U. S. AND SAHASRABUDHE, M. B.—(1957) *Brit. J. Cancer*, **11**, 482.
SAHASRABUDHE, M. B.—(1958) *Nature*, **182**, 163.
SICE, JEAN (1954) *J. org. Chem.*, **19**, 70.
VAN VALS, G. H., BOSCH, L. AND EMMELOT, P.—(1956) *J. biol. Chem.*, **222**, 399.
WARAVDEKAR, S. S. AND RANADIVE, K. J.—(1957) *J. nat. Cancer Inst.*, **18**, 555.
WENNER, C. E. AND WEINHOUSE, S.—(1956) *J. biol. Chem.*, **219**, 691.